# Problem perception and problem regulation during online collaborative learning: what is important for successful collaboration?

**DOI:** 10.3389/fpsyg.2024.1351723

**Published:** 2024-09-09

**Authors:** Martin Greisel, Laura Spang, Kerstin Fett, Ingo Kollar

**Affiliations:** Educational Psychology, University of Augsburg, Augsburg, Germany

**Keywords:** collaborative learning, socially shared regulation, challenges, homogeneity, intensity, immediacy, videoconferencing

## Abstract

**Background:**

University students frequently prepare for exams or presentations in self-organized study groups. For this purpose, they often use videoconferencing software. During their collaboration, they need to regulate emerging problems to ensure effective learning. We suppose that regulation is facilitated when (1) the group perceives their regulation problems homogeneously, (2) they choose regulation strategies that have the potential to solve the problems immediately, and (3) they execute these strategies with sufficient intensity.

**Aims:**

We investigated which problems occur during online collaborative learning via videoconferencing and how homogeneity of problem perceptions, immediacy of the chosen strategies, and intensity of strategy use are related to regulation success.

**Sample:**

University students (*N* = 222) from two lectures in pre-service teacher education and educational sciences in 99 study groups.

**Methods:**

Students collaborated in a self-organized manner, that is, without a teacher present, to study the material of one lecture using videoconferencing software. After the collaboration, group members rated, individually, the intensity of different problems during collaboration, reported which strategies they used to overcome their biggest problem, and rated the success of their problem regulation, their satisfaction with their collaboration, as well as their learning gain. In addition, they answered a knowledge test.

**Results:**

We found that most students rated technical issues as their biggest problem. Multilevel modeling showed that homogeneous problem perception moderated by problem intensity—contrary to immediate and intensive strategy use—predicted successful problem regulation and satisfaction with the collaboration but not knowledge gain. Case analyses illustrate the assumed mechanism that a homogeneous problem perception facilitates socially shared regulation.

**Conclusion:**

We conclude that even in only slightly structured learning contexts, students might only need to jointly identify their problems, whereas the best possible regulation of these problems seems less relevant. Therefore, training students to foster regulation competencies might prioritize identifying problems.

## 1 Problem statement

Many students deliberately form self-organized small study groups, e.g., to prepare for exams. Taking positive effects of collaborative learning on knowledge acquisition found in the literature into account (e.g., Chen et al., 2018; Jeong et al., 2019; Kyndt et al., 2013; Springer et al., 1999), this is a sensible decision. However, collaborative learning unfortunately is not always effective (Al-Samarraie and Saeed, 2018; Nokes-Malach et al., 2015; Weinberger et al., 2012). In fact, students may be confronted with a variety of problems during collaboration that may hinder effective learning (Al-Samarraie and Saeed, 2018; Järvenoja et al., 2013). Only if the group is able to regulate these problems successfully, collaborative learning is effective (Järvelä and Hadwin, 2013).

Yet, which problems occur during collaborative learning might be affected by how a meeting takes place. When groups cannot meet in person (e.g., at institutions for distance learning, in areas with large physical distances between students, or during times of a pandemic), collaborative learning typically happens online through videoconferencing tools such as Zoom or Skype. And indeed, when collaborating using videoconferencing software, technical issues such as a low stability of the network connection or difficulties in using the software functions arise and hinder effective collaborative learning (Belli, 2018; McCollum et al., 2019; Nungu et al., 2023; Rizvi and Nabi, 2021). This is also reflected in the finding that students’ intention to use videoconferencing depends on whether students believe that they have the necessary resources, access to relevant information, and helpdesk services available (Camilleri and Camilleri, 2022).

Besides mere technical issues, the technically mediated nature of communication such as a low visibility of non-verbal cues like facial expressions or gestures (Jeitziner et al., 2024) might complicate the collaboration further. For example, the phenomenon known as Zoom fatigue was attributed to the difficulty to keep track of nonverbal behavior, especially for women (Fauville et al., 2023). Furthermore, social presence which is known to affect active collaborative learning and student engagement (Qureshi et al., 2021), should evidently be lower in online meetings. Also, the extent to which videoconferencing allows for building trust and getting to know others is related to basic psychological need satisfaction, which, in turn, is associated with students’ behavioral and emotional engagement (Shi et al., 2024). Regarding, for example, building trust and the impression of others, eye-contact matters. If the video setup does not allow for the students to perceive eye contact, trust and the impression of others are negatively affected (Bohannon et al., 2013). Some of these problems might be mitigated when additional digital tools such as mapping tools are added to videoconferencing (Park et al., 2023). To summarize, collaborative learning through videoconferencing carries the risk of additional problems compared to learning collaboratively face-to-face.

However, not much is known about how the virtual context influences how students regulate problems occurring during collaborative learning, and respective findings were mixed: On the one hand, Capdeferro and Romero (2012) asked a relatively small sample of master students enrolled in a distance education university about their frustrations with online collaboration, which was text-based through discussion forums and email. Participants often reported to be frustrated due to various problems such as an imbalance in commitment, unshared goals, or communication difficulties. This frustration might indicate failed regulation of problems related to online collaboration. On the other hand, Tan et al. (2022) investigated how students used Microsoft Teams, applying an action research methodology. They found that students were able to collaborate relatively similar to face-to-face-settings.

From this state of the literature, we conclude the following research gaps: First, most of the literature reports students’ responses at an aggregated level, that is, general problems or satisfaction with online learning during a whole study semester or course (except Belli, 2018, who have, compared to the current research aim, a very narrow focus on emotional reactions to technical problems in single interactional units). It does not report problems at the level of a single session of collaborative learning. At this level, students might report a much more accentuated picture of problems which would be typically averaged out at the general level.

Second, most studies investigating collaborative learning via videoconferencing did not investigate how students regulated these problems. Only outcomes such as emotional reactions or satisfaction were examined, not processes of regulation. However, knowledge about regulation processes is necessary to inform support measures.

Third, self-organized groups or conditions similar to self-organized groups were not investigated. All studies collected data from students that collaborated based on teacher instruction. For example, the students from the two studies that investigated the outcomes of regulation (Capdeferro and Romero, 2012; Tan et al., 2022) collaborated across several weeks on a task that teachers specifically designed as an effective collaborative task. However, self-organized study groups differ in this regard by definition. Their engagement is voluntary, not scaffolded by teachers, and they are on their own during collaboration. Therefore, it should be only up to the students how beneficial for learning their meeting will be.

In conclusion, self-organized study groups need the skills to regulate problems during a session of collaborative learning, but we do not know yet which problems occur in these sessions and how groups can regulate them successfully. Addressing these research gaps is important because it will inform training. As self-organized study groups are unassisted, they need to be equipped with all necessary regulation skills themselves. Consequently, they need to acquire these skills before they enter a self-organized study group. However, for research to be able to develop adequate training, we first need to know how self-organized study groups regulate the problems they encounter. Therefore, this study focuses on which problems occur and how problems are regulated in virtual collaborative learning through videoconferencing.

## 2 Regulation of problems in collaborative learning

According to Chen et al. (2018, p. 800), “collaborative learning […] emphasizes that knowledge is co-constructed through social interaction. It is a learning situation in which two or more students learn together to achieve a common goal or solve the task at hand, mostly through peer-directed interactions.” Usually, collaborative learning is instructed by a teacher. However, students also self-organize and form study groups on their own initiative without teacher support. Therefore, they voluntarily meet outside the classroom or outside the regular virtual class context to study based on their own goals. Most likely, this happens to prepare for exams. In the present article, we use the conceptualization of “collaboration in self-organized study groups as an instance of (socially) self-regulated learning (Järvelä and Hadwin, 2013) that requires groups to make decisions on their own learning process (e.g., concerning questions such as when and how long to meet, how to approach comprehension problems, or what technology to use during collaboration)” by Melzner et al. (2020, p. 150).

Based on previous research (e.g., Järvenoja et al., 2019; Melzner et al., 2020), problems in collaborative learning (and consequently also in self-organized collaborative learning) can be divided into at least the following categories: (a) comprehension problems (e.g., learners may have difficulty understanding the task), (b) coordination problems (e.g., learners may have different goals for learning together), (c) motivation problems (e.g., the learning contents may be perceived as not useful), and (d) resource-related problems (e.g., a digital tool might lack a necessary function). For self-organized collaborative learning to be successful, groups should be able to cope with such problems successfully.

**Figure 1:**
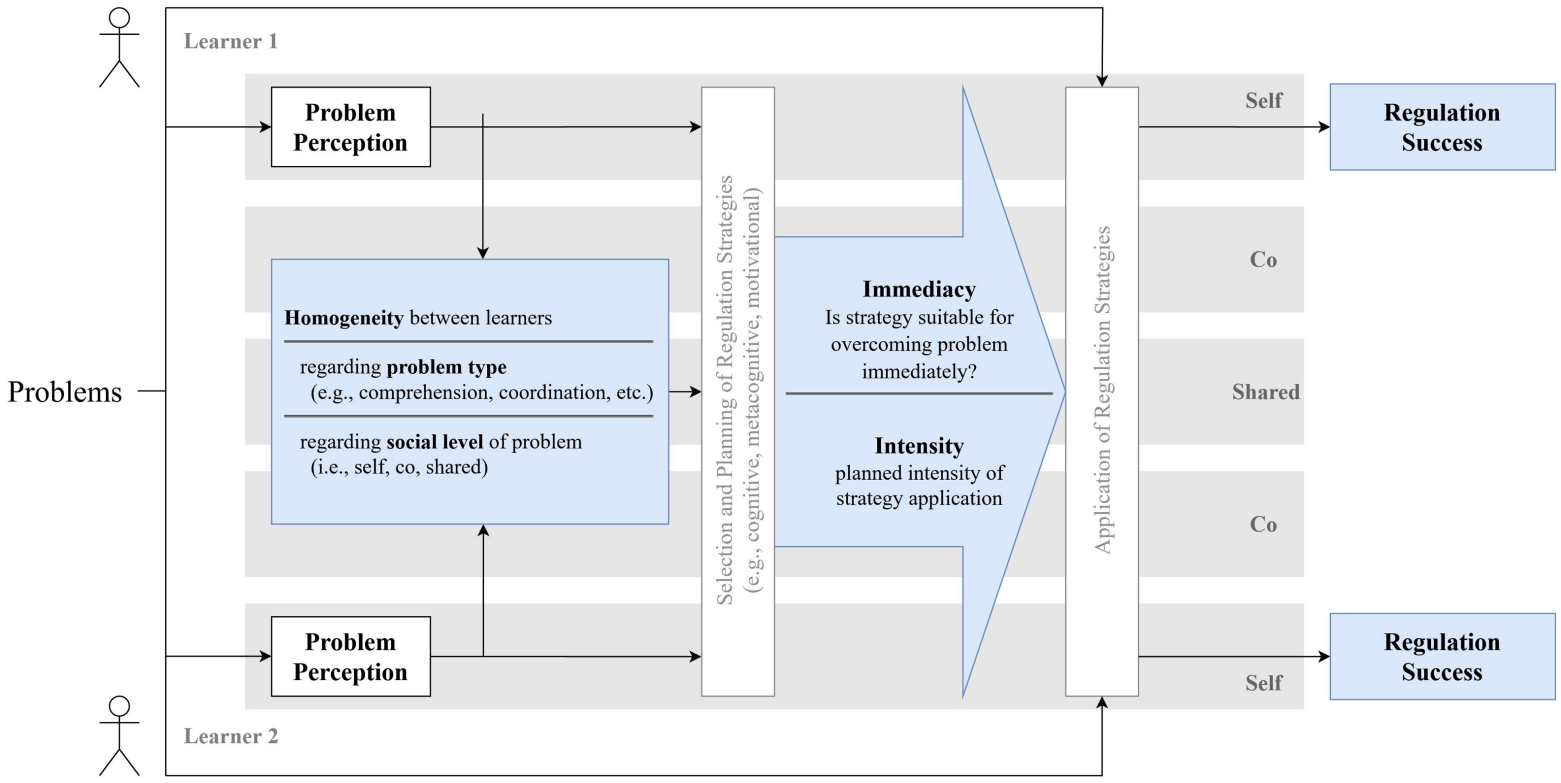
Theoretical model of the regulation of problems during collaborative learning (visualization inspired by Wecker and Fischer, 2014). Concepts in boldface are measured in the present study. Adapted by permission from Springer Nature: IJCSCL. Regulating self-organized collaborative learning: The importance of homogeneous problem perception, immediacy and intensity of strategy use (Melzner et al., 2020).

To conceptualize the processes involved in this problem regulation, Melzner et al. (2020) developed a heuristic process model (see Figure 1) following models of (socially shared) regulated learning (e.g., Hadwin et al., 2018; Winne and Hadwin, 1998). Based on these models, metacognitive processes are especially crucial for the successful regulation of problems in collaborative learning. By aid of such processes, students (1) perceive and classify these problems. Based on the assessment of a problem, they initiate a reaction to ensure that the goal is achieved despite the problem at hand. For this purpose, students (2) select a strategy to address the problem and (3) execute this strategy with a certain intensity. Along with Melzner et al. (2020), we assume that these three processes (problem perception, choice of regulation strategy, intensity of strategy execution) should predict success in the regulation of problems that occur during collaborative learning. The different parts of the model proposed by Melzner et al. (2020) are more deeply elaborated in the following.

### 2.1 Homogeneity of problem perception

At the beginning of the regulation process, learners perceive and classify a given problem (see Figure 1). Different group members may arrive at different problem assessments. Divergences can basically refer to two dimensions: On the one hand, students may perceive problems of varying types (see, e.g., Järvenoja et al., 2013). For example, while one learner may perceive a comprehension problem to be present (e.g., the subject matter is perceived as too difficult), another learner may identify a motivational problem (e.g., the subject matter is not useful for practical application). On the other hand, there may also be disagreement about the social level at which the problem is located. Using the classification of Järvelä and Hadwin (2013), it can be distinguished whether a learner is affected themselves (self-level), whether the problem affects individual other group members (co-level), or whether the whole group is affected (socially shared level). The homogeneity of the problem perception is thus to be understood in terms of (a) the type of problem and (b) the question who is affected by the problem.

We suspect that diverging perceptions of the problem within the group make collaborative learning more difficult. The reasoning is straightforward: From a perspective of regulated learning (Winne and Hadwin, 1998), students realize that there is a problem if the outcomes (= products) of learning operations do not match their standards, that is, learning does not proceed as it should. As a reaction, they modify their operations (= control) to address this discrepancy. However, if different students assess different aspects of their collaboration or employ different standards, they see different problems, and, consequently, aim to modify their learning in different directions. Therefore, individual group members are less likely to coordinate their regulation efforts, just as a group of people walking into different directions has trouble to agree on a common pathway. If, in contrast, group members share a similar perception of problems that need to be regulated, this might help them to regulate the problem (Borge et al., 2018; Splichal et al., 2018). Indeed, Melzner et al. (2020) found that the more homogeneous students perceived their problems, the more satisfied they were with their collaboration.

### 2.2 Immediacy of regulation strategy use

Next, learners select a strategy for the regulation of the previously perceived problem (see Figure 1). Models of self-regulated learning (e.g., Winne and Hadwin, 1998; Zimmermann and Moylan, 2009) assume that at this point, the choice of a strategy that fits the learning goal is crucial. Collaborative learning groups that face a variety of problems need to use different strategies since not every strategy is supposed to be equally well suited to achieve a particular goal (e.g., Engelschalk et al., 2016; Malmberg, et al., 2015). In our view, a similar assumption may be made regarding the fit between an emerging problem and the chosen strategy for its regulation (e.g., Engelschalk et al., 2016). However, previous research has hardly made statements about what is meant by *fit*.

In order to operationalize fit, we have proposed the concept of *immediacy* (Melzner et al., 2020): A strategy is considered to be immediate for a problem if it is in principle possible to actually solve the problem without further strategies necessary when the respective strategy is executed optimally. An example of an immediate strategy would be to switch off cell phones when the group is distracted by incoming messages during learning. An example of a non-immediate strategy would be if learners make themselves aware of the importance of the exam they are preparing for in order to motivate them to continue learning despite the incoming messages. This strategy would only allow learners to continue learning despite the presence of the problem. However, it would not eliminate the source of distraction and thus would not immediately make the problem disappear.

Thus, for the operationalization of fit, a theoretical assignment of strategies to problems as immediate or non-immediate was proposed by Melzner et al. (2020) and was found to predict self-organized, offline groups’ satisfaction with their collaboration. In addition, prior research often investigated the fit of various strategies for different learning situations via expert ratings (e.g., Artelt et al., 2009; Bäulke et al., 2018; Fett et al., 2021; Steuer et al., 2019; Waldeyer et al., 2019). For instance, Waldeyer et al. (2019) asked experts to rate the effectiveness of several resource strategies for the regulation of 60 resource demanding situations that learners might face during their studies. For 36 out of these 60 situations, experts agreed on one strategy as the most fitting strategy. Further, students who selected the same strategies as the experts for a given situation performed better in an exam. In comparison, Fett et al. (2021) asked experts from computer-supported collaborative learning and self-regulated learning research to rate how immediate strategies regulate a given problem in a collaborative learning setting. As a proof of concept, experts assigned strategies to problems very selectively and highly agreed on the immediacy for a large proportion of problem-strategy-pairs.

### 2.3 Intensity of the execution of the regulation strategy

To be effective, the selected strategy must be applied in the next step (see Figure 1). Depending on the severity of the problem, however, a single application of the strategy may not be sufficient to achieve the desired effect. For example, if learners who are bored by the learning material think only briefly about their goals for the future, this may have little effect on their motivation to devote effort toward understanding the material. However, if they work intensively on how the material will help them achieve their own goals, this should increase their motivation. We therefore assume that the intensity of strategy use is positively related to regulation success. However, not only the intensity of using immediate strategies should be relevant, since also the use of non-immediate strategies might increase regulation success, even if the specific problem is not solved that way. In line with this reasoning, it is not surprising that findings regarding the effects of regulation intensity on individual and group outcomes are mixed (Eckerlein et al., 2019; Melzner et al., 2020; Schoor and Bannert, 2012). Thus, more research is needed to clarify its influence on regulation success.

### 2.4 Operationalizing regulation success in collaborative learning

Once the regulation process is executed in accordance with the process model depicted in Figure 1, it should be successful. Yet, regulation success may be conceptualized and measured in various ways (e.g., Melzner et al., 2020; Noroozi et al., 2019; Zimmermann and Moylan, 2009). In this paper, we focus on four different conceptualizations: (1) successful regulation of the biggest problem which occurred during the collaborative learning (i.e., the extent to which the problem is overcome), (2) satisfaction with the collaboration, and (3) the subjective and (4) objective learning success resulting from the group learning session. So far, only satisfaction has been empirically investigated in this context (e.g., Bellhäuser et al., 2019; Melzner et al., 2020). For example, Bellhäuser et al. (2019) experimentally examined how group composition regarding group members’ extraversion and conscientiousness affected their rating of how satisfied they were with the quality of their collaboration.

Yet, not much is known about how problem perception, immediacy and intensity of strategy use contribute to further measures of regulation success. It can be assumed that effects might differ in strength because the suggested variables differ in how proximate they are to regulation during collaborative learning: Successfully overcoming problems could be considered the prime and most direct outcome of regulation. Satisfaction with the collaboration is probably based on more variables besides successful problem regulation, for example task difficulty (Kirschner et al., 2009), task design, or group members’ preference for group work (Shaw et al., 2000), or their achievement goals (Greisel et al., 2023), but it should still be linked closely to the regulation process (Melzner et al., 2020; Bellhäuser et al., 2019). Subjective knowledge gain, in turn, is more distal as it should also be affected by the effectiveness of the employed cognitive learning strategies, the quality of the task and the learning material, the learning goals, etc. Moreover, objective knowledge is also not dependent on the quality of the collaboration alone, as students can memorize learning content also outside of a collaborative setting. Nonetheless, groups’ successful problem solving has repeatedly been linked with knowledge gain and performance outcomes empirically (e.g., Chen et al., 2018; Kirschner et al., 2011).

## 3 Research questions and hypotheses

We briefly summarize the research gaps mentioned so far: First, it is unclear which problems students in self-organized study groups experience when they collaborate using a videoconferencing tool *without teacher guidance*. Second, it is an open question to what extent the three processes proposed by Melzner et al. (2020, homogeneity of problem perceptions, immediacy of strategy use, and intensity of strategy use) predict successful regulation in collaborative online settings. Third, little is known about whether these three processes are differentially predictive of the four conceptualizations of regulation success described above. Therefore, our study aims to answer the following research questions:

- Which problems do students experience to which extent while collaborating online via videoconferencing without teacher guidance?
- How are homogeneity of problem perceptions, immediacy of strategy use, and intensity of strategy use associated with successfully overcoming problems during online collaboration via videoconferencing, satisfaction with the collaboration, subjective knowledge gain, and objective knowledge?

The first research question is investigated exploratorily as the current state of knowledge does not allow for predicting outcomes, whereas regarding the second research question, we formulated the following confirmatory hypotheses:

1. The more homogeneously learners perceive problems within their groups, the more positive are the results on different measures of regulation success.
2. Learners who use immediate strategies to regulate their problems achieve more positive results on different measures of regulation success than learners who use only non-immediate strategies.
3. The more intensively learners apply regulation strategies, the more positive are the results on different measures of regulation success.

## 4 Method

### 4.1 Sample

University students (*N* = 222) from two basic psychological lectures in German language within the majors educational sciences (29%) and teacher training (70%) from a university in Southern Germany learned collaboratively and anonymously answered an online questionnaire afterwards. They had an average age of 22 years (*M* = 21.84, *SD* = 4.39, 83% female), were on average in the third semester of their current study subject (*M* = 2.78, *SD* = 1.50) and in their third university semester overall (*M* = 3.34, *SD* = 2.57). Participating in this session of collaborative learning was voluntary (i.e., not necessary for being admitted to the exam at the end of the course). We advertised it as a good chance to learn the subject matter relevant to the exam. However, students experienced no disadvantages if they did not participate in the collaborative learning session or the study. Individual data were not provided to the lecturers of the courses.

Participants self-assigned into 99 small groups of three persons on average (*M* = 2.92, *SD* = 0.27, 11 groups with two persons, 88 groups with three persons, self-reported), but not all members of each group participated in the study. Therefore, we have data from *M* = 2.24 (*SD* = 0.86) persons per group only. In detail, 26 groups were represented by one person, 24 groups by two persons, 48 groups by three persons, and one group by four persons (all rows from the group with four persons seemed to represent distinct persons, therefore we decided to keep it though no group reported to have 4 persons). The data from the 26 groups which were represented in our data by a single person had to be excluded from all analyses that included the calculation of homogeneity of problem perception as this is possible only for groups with data of two or more learners.

### 4.2 Procedure

The study was embedded in two large lectures that mainly consisted of weekly uploaded recordings of PowerPoint-presentations (i.e., slides with audio-recorded lecturer voice) that were provided for individual, asynchronous studying during the summer term 2020 after the onset of the COVID-19 pandemic. One session of collaborative learning replaced the regular recorded lecture in the respective week. The subject matter of this week was not repeated or discussed in a later session, that is, we employed no flipped classroom pedagogy. Thus, learners acquired the knowledge only in a self-organized fashion. Learners were instructed to meet online at a time suitable for all group members using a videoconferencing software of their choice to study the lecture content on their own. Students collaborated for *M* = 90.6 (*SD* = 40.6) minutes (self-report). Only three students indicated a studying time of less than 30 min. As learning material, the regular presentation slides for this session (without audio) were provided alongside two excerpts from a textbook, each about one page long. Topics were the ICAP model of learning activities (Chi and Wylie, 2014) and the multi-store model of memory (Atkinson and Shiffrin, 1968). We did not structure or scaffold students’ collaborative learning with additional instructions. We only provided them with the following tasks: “The goal of the group work is to work out the slide contents as well as possible together with your group members. You are welcome to use the additional texts provided.” In addition, students were told to record the results of their group work in a shared concept map. Yet, besides this, learners were free to decide in which way, that is, with which activities or tools, they wanted to work on the topic. This instructional design should mimic learning in a self-organized study group as closely as possible. For learners who were not familiar with an online tool suitable to produce a concept map, we recommended www.mindmeister.com and provided a short tutorial video explaining all functions necessary to accomplish the task.

After the study meeting, participants were asked to individually answer an online questionnaire. The questionnaire was advertised as containing a knowledge test for which students would receive immediate feedback regarding correct and incorrect answers. The questions were comparable to the ones in the final exam in the corresponding lectures, so taking the test would be a good chance to practice for the “real” exam.

### 4.3 Measures

**Figure 2:**
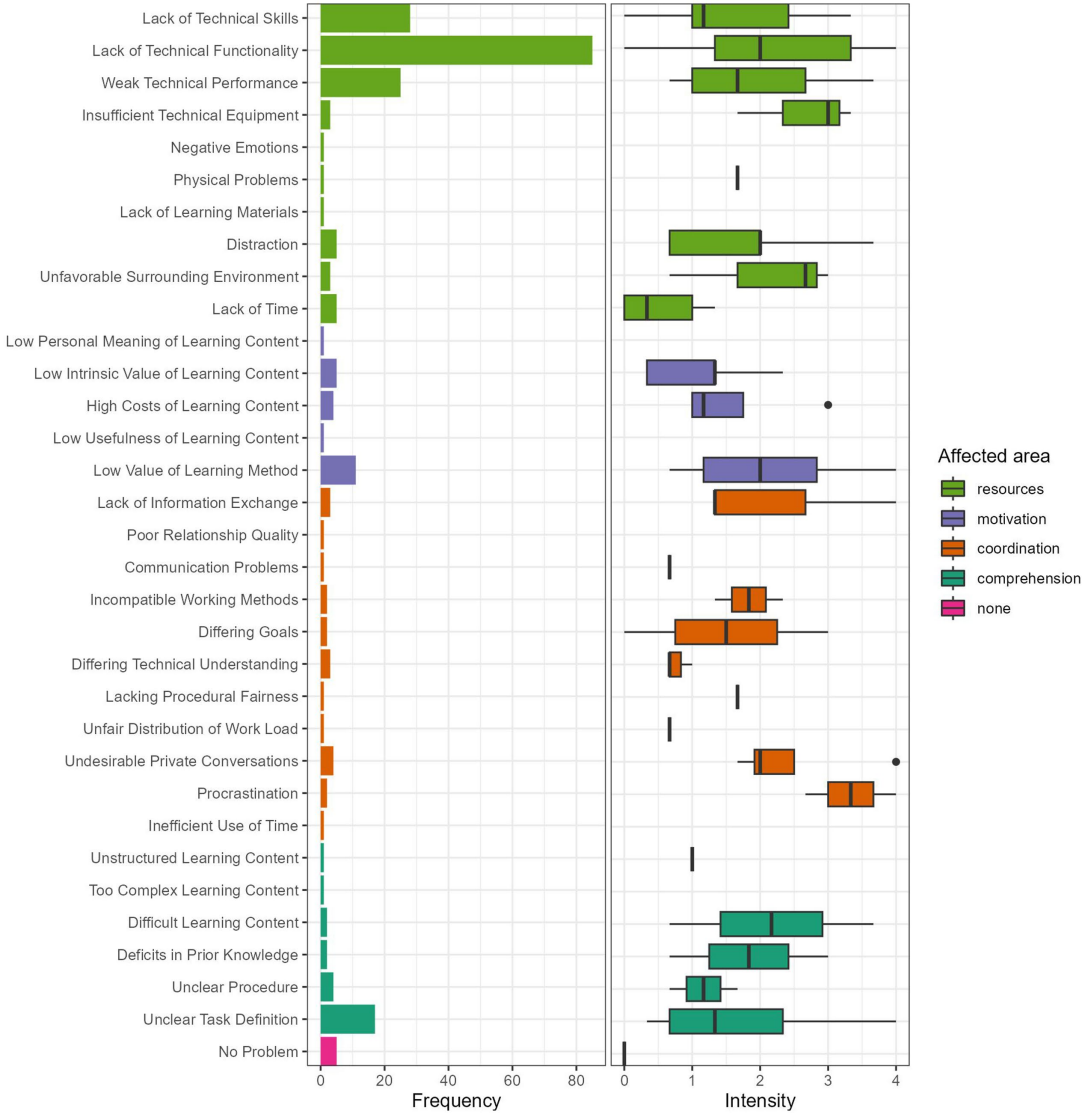
Frequency and intensity of problems selected as biggest problem during collaborative learning. Left panel shows how often participants selected a certain problem as biggest problem. Right panel displays boxplots of the problem intensities (only for those participants who selected the respective problem as biggest problem). Single vertical lines without surrounding box result if only one person selected this problem as biggest problem. Empty lines result if participants did select the respective problem as biggest problem but did not rate its intensity.

To measure the *prevalence of problems during collaborative learning*, we developed a questionnaire with 32 different problems represented by three items each. Each item had to be rated on a Likert-scale from 0 = *did not occur/no problem* to 4 = *big problem*. Based on problem typologies and theoretical classifications in the literature (e.g., Järvenoja et al., 2013; Koivuniemi et al., 2017), our questionnaire covered four broad categories of problems: comprehension-, coordination-, motivation-, and resource-related problems (see Figure 2 for a complete list of individual problems). For example, for the problem of “low value of learning method” (a motivational problem), a sample item was “Single/multiple group members did not find group work to be a useful learning method in the given situation.” Cronbach’s alpha was *M* = 0.79 (*SD* = 0.07; [0.59–0.92]) on average. After rating all problems, participants selected one of them as the biggest problem they encountered during the learning session.

To validate the factor structure of this problem scale, we conducted an extensive series of confirmatory factor analyses. As preparation, we first grouped items which are theoretically at least somewhat similar into different sets of similar items. An item could be part of several sets. This grouping was necessary because a confirmatory factor analysis with 32 latent variables and 96 indicators would not have been methodologically sound, given the sample size, the degrees of freedom, and the number of parameters to estimate. Then, we conducted multiple confirmatory factor analyses for each of these groups of similar items. Thereby, we compared the hypothesized factor structure (3 items per factor) to other theoretical plausible factor structures. Most of the time, this was a unidimensional model and models with slightly more or less factors, sometimes also with second order factors. In the end, we compared the model fits of these models to decide whether the hypothesized factors with three items per factor were distinguishable from each other, and whether the hypothesized factor solutions had the best fit to the data. This was the case for all problems, hence we decided to keep the intended factor structure with 32 problems and three items each.

To determine the *homogeneity of the within-group perceptions regarding the type of problem*, we calculated the deviation of each person’s rating from the average ratings of the remaining group members. We did this for each problem separately and then determined the average deviation across all problems. To transform the deviation into a measure of homo- instead of heterogeneity, we multiplied it by −1. Thus, a value of 0 represents perfect homogeneity of problem perceptions, whereas the more negative the value is, the less homogeneous the perception was. To determine the *homogeneity of within-group problem perceptions regarding the social level*, we used three items measuring the extent to which the biggest problem affected the self-, co-, or shared level on a five-point Likert-scale from 1 = *not at all true* to 5 = *completely true*. The items were “The mentioned problem had effects on my personal learning process” (self), “The mentioned problem had effects on single other group members’ learning process” (co), and “The mentioned problem had effects on the whole group’s learning process” (socially shared). The ratings for each item were dichotomized by median split (*Md_self_* = 2, *Md_co_* = 2, *Md_shared_* = 3), resulting in a zero–one-coding. Then, groups were coded as being homogeneous regarding the social level of problem perception when the social level at which they located the biggest problem matched the respective ratings of each other group member. For example, a group’s problem perception was considered to be homogeneous when one person located the problem only at the self-level, while the two other group members located the problem only at the co-level.

To measure *immediacy and intensity of strategy use*, we asked participants to name the strategies they used to regulate the problem they marked as the biggest one at the self-, co-, and socially shared level in an open answer format (self-level: “What did you think/do/say for yourself to get to grips with the biggest problem?”; co-level: “What did you think/do/say for others to get to grips with the biggest problem?” and “What did others do/say for you to get to grips with the biggest problem?”; socially shared level: “What did you think/do/say as a group to get to grips with the biggest problem?”). These answers were segmented into single regulation strategies (interrater-agreement 90–91%). Then, each strategy was classified as one out of 27 possible types of strategies (for a list, see Melzner et al., 2020). Interrater reliability of two independently coding, trained student research assistants was sufficient (Gwet’s AC1 = 0.73). Next, each strategy was assigned a value which expressed the extent to which experts considered this strategy to be immediate for the selected biggest problem. The expert ratings stem from another study which asked experts from CSCL and self-regulation to rate how immediately different regulation strategies solve a given problem on a scale from 0 to 4 (Fett et al., 2021). To determine the intensity of strategy use, we added up the number of valid regulation strategies reported at all social levels.

To measure *successful problem regulation*, we adapted three items from Engelschalk et al. (2016) (e.g., “During group learning, we got the biggest problem under control.”). Each item had to be rated on a Likert-scale from 1 = *not at all true* to 5 = *completely true*. Cronbach’s alpha was 0.96.

*Satisfaction with the collaboration* was measured by five items from the German version of the Satisfaction with Life Scale (SWLS; Glaesmer et al., 2011), which we adapted to the group learning context (e.g., “Our group work was excellent.”). Each item employed a 5-point Likert scale ranging from 1 = *not at all true* to 5 = *completely true*. Cronbach’s alpha was 0.92.

We assessed *subjective knowledge gain* by using six adapted items from the Training Evaluation Inventory (TEI; Ritzmann et al., 2014). Knowledge gain with regard to the ICAP model (Chi and Wylie, 2014) and knowledge gain with regard to the multi-store model of memory (Atkinson and Shiffrin, 1968) were measured separately by three items each (e.g., “I have the impression that my knowledge on the ICAP-Model/the multi-store model of memory has expanded on a long-term basis”) on a 5-point Likert-scale from 1 = *not at all true* to 5 = *completely true*. Cronbach’s alpha was 0.92.

As a measure of *objective knowledge*, we mimicked a typical standardized lecture exam: We constructed eight multiple choice questions with four dichotomous answer alternatives each (four questions for each theory). Sample answer options were “The production of new knowledge could be realized through the exchange of different perspectives of different learners” (ICAP) and “Sensory information is stored for a very short period of time but is then overwritten by new information” (multi-store model of memory). To validate this test, we inspected item difficulties and conducted a distractor analysis. We removed items which were correctly answered by more than 90% of the sample as these items did not differentiate between high and low scorer. In addition, we had to remove one item which was not clearly correct or false and one item which had an inverse relation to the total score even after reversing intentionally inverted items. As the distractor analysis indicated, the remaining items differentiated well between the upper, middle, and lower percentile of total scores. Corrected item-scale-correlations indicated that the items measured, as intended and typical for a knowledge test, different aspects of knowledge regarding the two theories and not a single homogeneous latent construct. Then, we calculated separate mean scores (= proportion of right answers) for each theory, and, finally, we averaged these two scores to get one total test score.

### 4.4 Analysis strategy

As preliminary analyses, we inspected descriptive statistics of predictor and criterion variables (see Table 1 and Table 2). In addition, we tested whether the nesting of students in courses needed to be considered. As a MANOVA showed no significant differences between the two courses in the dependent variables, *F*(1, 216) = 0.73, *p* = 0.572, we did not consider the course level in the further analyses. In contrast, the ICCs (see Table 1) indicated that belonging to a specific study group explained a considerable proportion of the variance of each dependent variable, thus we had to take the clustering of students in groups into account.

**Table 1:** Multilevel modeling of four different measures of regulation success.

	Successful problem regulation					Satisfaction with collaboration					Subjective knowledge gain					Objective knowledge				
Predictors	*b*	CI	*p*	std. *β*	std. CI	*b*	CI	*p*	std. *β*	std. CI	*b*	CI	*p*	std. *β*	std. CI	*b*	CI	*p*	std. *β*	std. CI
(Intercept)	4.31	3.58 to 5.04	**<0.001**	0.10	−0.05 to 0.25	4.56	4.06–5.07	**<0.001**	0.08	−0.07 to 0.22	4.01	3.36–4.66	**<0.001**	0.03	−0.13 to 0.20	0.75	0.67–0.84	**<0.001**	−0.01	−0.18 to 0.16
Homogeneity	0.49	−2.72 to 3.70	0.762	0.58	−0.38 to 1.53	0.55	−1.72 to 2.82	0.633	0.50	−0.40 to 1.41	−0.84	−3.77 to 2.08	0.570	−0.16	−1.17 to 0.85	0.19	−0.19 to 0.57	0.327	0.43	−0.62 to 1.47
Problem intensity	0.06	−0.21 to 0.33	0.654	−0.32	−0.47 to −0.18	−0.00	−0.18 to 0.18	0.996	−0.27	−0.40 to −0.13	−0.12	−0.35 to 0.12	0.324	−0.26	−0.41 to −0.11	0.01	−0.02 to 0.04	0.574	0.16	0.00 to 0.32
Homogeneity quadratic	2.05	−2.61 to 6.71	0.386	0.84	−1.07 to 2.76	0.13	−3.14 to 3.41	0.938	0.07	−1.72 to 1.86	−2.38	−6.60 to 1.83	0.266	−1.13	−3.12 to 0.87	0.24	−0.32 to 0.80	0.396	0.90	−1.19 to 2.99
Homogeneity cubic	0.52	−1.30 to 2.35	0.572	0.31	−0.78 to 1.40	−0.23	−1.50 to 1.04	0.718	−0.19	−1.20 to 0.83	−1.37	−3.00 to 0.26	0.098	−0.94	−2.07 to 0.18	0.11	−0.11 to 0.33	0.324	0.59	−0.59 to 1.780
Homogeneity social	0.10	−0.26 to 0.46	0.589	0.04	−0.10 to 0.18	0.00	−0.26 to 0.26	0.992	0.00	−0.14 to 0.14	−0.09	−0.44 to 0.25	0.586	−0.04	−0.20 to 0.11	−0.03	−0.07 to 0.01	0.188	−0.10	−0.26 to 0.05
Immediacy	0.08	−0.05 to 0.20	0.232	0.08	−0.05 to 0.21	0.02	−0.06 to 0.11	0.625	0.03	−0.09 to 0.15	0.01	−0.10 to 0.12	0.870	0.01	−0.12 to 0.14	−0.01	−0.02 to 0.01	0.480	−0.05	−0.19 to 0.09
Regulation intensity	0.03	−0.03 to 0.09	0.290	0.07	−0.06 to 0.20	0.04	−0.00 to 0.08	0.053	0.12	−0.00 to 0.24	0.03	−0.02 to 0.08	0.215	0.08	−0.05 to 0.22	0.00	−0.00 to 0.01	0.348	0.07	−0.08 to 0.21
Homogeneity * Problem intensity	0.80	0.29 to 1.30	**0.002**	0.25	0.09–0.42	0.40	0.06 to 0.75	**0.022**	0.18	0.03 to 0.33	0.20	−0.24 to 0.64	0.371	0.08	−0.09 to 0.24	−0.02	−0.08 to 0.04	0.600	−0.05	−0.23 to 0.13
**Random effects**																				
σ2	0.84					0.37					0.59					0.01				
τ00	0.07 *GrNr*					0.07 *GrNr*					0.14 *GrNr*					0.00 *GrNr*				
ICC	0.08					0.17					0.19					0.12				
N	74 *GrNr*					74 *GrNr*					74 *GrNr*					74 *GrNr*				
Observations	194					198					198					197				
Marginal *R*^2^/Conditional *R*^2^	0.195/0.258					0.285/0.403					0.147/0.307					0.048/0.159				

*p-values in bold face < 0.05.*

**Table 2:** Means, standard deviations, and correlations.

Variable	*M*	*SD*	1	2	3	4	5	6	7	8
1. Homogeneity problem type	−0.46	0.30								
2. Homogeneity social level	0.21	0.41	0.04							
3. Immediacy	1.27	1.09	0.04	−0.03						
4. Regulation intensity	3.99	2.39	−0.02	0.10	0.05					
5. Problem intensity	1.86	1.16	−0.39**	−0.08	0.06	0.14*				
6. Successful problem regulation	4.12	1.07	0.27**	0.11	0.08	0.08	−0.38**			
7. Satisfaction with the collaboration	4.12	0.84	0.46**	0.06	0.04	0.09	−0.42**	0.53**		
8. Subjective knowledge gain	3.76	0.89	0.27**	−0.01	−0.01	0.04	−0.28**	0.33**	0.33**	
9. Objective knowledge	0.74	0.12	0.02	−0.11	−0.00	0.14*	0.08	0.03	−0.06	0.07

^*^p < 0.05; ^**^p < 0.01.

To answer Research Question 1, we investigated descriptively which problems participants selected as biggest problems and as how severe they assessed them. To answer Research Question 2, we conducted multilevel regression analyses with the REML estimator to account for the two-level structure (students in study groups) and covariations between predictor variables. Therefore, we used R [4.2.2] (R Core Team, 2022) with the package lme4 [1.1–31] (Bates et al., 2015) and lmerTest [3.1–3] (Kuznetsova et al., 2017). As an inspection of the scatter plots for the bivariate relations indicated, the relation between homogeneity regarding the problem type and the dependent variables described are more quadratic or cubic than linear curve. Therefore, we added quadratic and cubic terms for homogeneity of problem type to account for this. As problem intensity logically determines the possible variance of homogeneity regarding the problem type within each group, we controlled for the interaction of problem intensity with homogeneity.

To complement the quantitative analyses, we described the answers from two groups. This qualitative illustration serves two purposes. First, it illustrates the interaction effect found in the quantitative analyses. Second, it sheds light on the theoretically assumed mechanism how homogeneity of problem perceptions facilitates problem regulation. Therefore, we chose two contrasting cases which prototypically represented opposing values in homogeneity and intensity of the biggest problem.

## 5 Results

### 5.1 Descriptives and bivariate correlations

A minority of participants (21%) located the biggest problem at the same social level within their groups (see Table 2). The problems where this happened relative to the total number of notions most frequently were “unclear task definition” and “low value of learning method” (for a detailed list, see Table 3). Regarding immediacy, 71% of the participants applied at least one immediate regulation strategy to remedy the biggest problem. Regardless of the type, about four strategies were reported on average. Both successful problem regulation and satisfaction with the collaboration were rated on average with *M* = 4.12 (successful problem regulation: *SD* = 1.07; satisfaction: *SD* = 0.84) on a scale from 1 to 5 and consequently estimated to be rather high, while subjective knowledge gain was appraised a bit lower (*M* = 3.76, *SD* = 0.89). Of all test questions measuring objective knowledge, 74% were solved correctly on average (*SD* = 0.12). Predictor variables were not significantly associated with each other, except for problem intensity with homogeneity, *r* = −0.39, *p* < 0.01, and regulation intensity, *r* = 0.14, *p* < 0.05. In addition, satisfaction with the collaboration was associated with successful problem regulation, *r* = 0.53, *p* < 0.01, and subjective knowledge gain, *r* = 0.33, *p* < 0.01, which were also correlated with each other, *r* = 0.33, *p* < 0.01. Consequently, all subjective measures for regulation success were associated with each other. Correlation analyses between the predictor and outcome variables showed that only content-related homogeneity of problem perception was associated with satisfaction with the collaboration, *r* = 0.46, *p* < 0.01, successful problem regulation, *r* = 0.27, *p* < 0.01, and subjective knowledge gain, *r* = 0.27, *p* < 0.01. Objective knowledge was only related to regulation intensity, *r* = 0.14, *p* < 0.05.

**Table 3:** Cross-tabulation of the biggest problem and homogeneity of within-group problem perceptions regarding the social level.

Biggest problem	Social homogeneity		Total
	No	Yes	
No problem	4 2%	0 0%	4 2%
Unclear task definition	9 4.5%	7 3.5%	16 8%
Unclear procedure	3 1.5%	0 0%	3 1.5%
Deficits in prior knowledge	1 0.5%	0 0%	1 0.5%
Difficult learning content	2 1%	0 0%	2 1%
Too complex learning content	0 0%	0 0%	0 0%
Unstructured learning content	1 0.5%	0 0%	1 0.5%
Inefficient use of time	0 0%	0 0%	0 0%
Lack of time	2 1%	1 0.5%	3 1.5%
Unfair distribution of work load	0 0%	0 0%	0 0%
Lacking procedural fairness	1 0.5%	0 0%	1 0.5%
Differing technical understanding	3 1.5%	0 0%	3 1.5%
Differing goals	1 0.5%	1 0.5%	2 1%
Incompatible working methods	2 1%	0 0%	2 1%
Communication problems	1 0.5%	0 0%	1 0.5%
Poor relationship quality	0 0%	0 0%	0 0%
Lack of information exchange	2 1%	0 0%	2 1%
Unfavorable surrounding environment	2 1%	1 0.5%	3 1.5%
Distraction	3 1.5%	0 0%	3 1.5%
Undesirable private conversations	3 1.5%	0 0%	3 1.5%
Lack of learning materials	0 0%	0 0%	0 0%
Physical problems	0 0%	0 0%	0 0%
Low value of learning method	4 2%	5 2.5%	9 4.5%
Low usefulness of learning content	0 0%	0 0%	0 0%
High costs of learning content	3 1.5%	0 0%	3 1.5%
Low intrinsic value of learning content	4 2%	1 0.5%	5 2.5%
Low personal meaning of learning content	0 0%	0 0%	0 0%
Procrastination	0 0%	1 0.5%	1 0.5%
Negative emotions	0 0%	0 0%	0 0%
Insufficient technical equipment	3 1.5%	0 0%	3 1.5%
Weak technical performance	21 10.6%	4 2%	25 12.6%
Lack of technical functionality	62 31.3%	14 7.1%	76 38.4%
Lack of technical skills	20 10.1%	6 3%	26 13.1%
Total	157 79.3%	41 20.7%	198 100%

### 5.2 Research question 1: Problems

Regarding Research Question 1, most students (*n* = 102) selected technical problems as their biggest problem during collaboration (Figure 2; Table 3). These were mostly centered around the recommended mind mapping-software. Only few students had insufficient equipment (*n* = 3). However, if this occurred, the problem was rather severe. Some students (*n* = 9) considered a low value of the learning method as their biggest problem, which had a medium intensity most of the time. The same applied to unclear task definition (*n* = 16; see Figure 2 and Table 3 for a complete list).

### 5.3 Research question 2: Predicting regulation success

**Figure 3:**
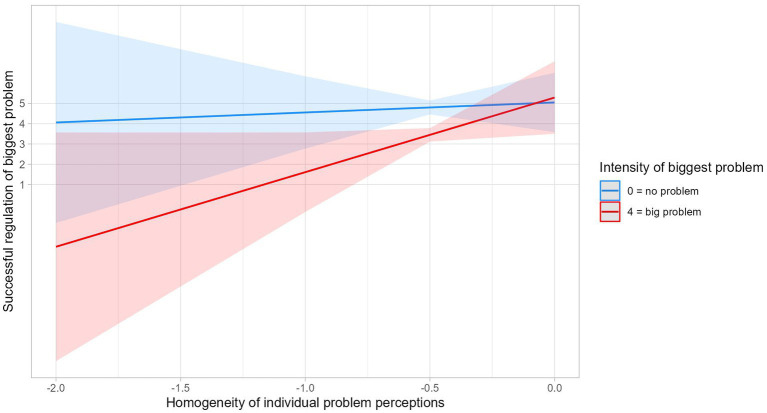
Interaction of homogeneity of individual problem perceptions and the intensity of the biggest problem.

Regarding Research Question 2, we calculated regression models (see Table 1). All hypotheses concerning main effects were not supported: Neither homogeneity regarding the problem type nor the social level, nor immediacy of strategy us, nor regulation intensity were associated with regulation success. However, explorative analyses showed that the interaction of homogeneity regarding the problem type and the problem intensity was a significant predictor of successful regulation of the biggest problem, *β* = 0.25, *p* = 0.002, and satisfaction with the collaboration, *β* = 0.18, *p* = 0.022. That is, for students who perceived the biggest problem in their group as severe, the more they perceived the problems similar to their group, the more successful they regulated the biggest problem and the more satisfied they were with the collaboration. However, for students who perceived the biggest problem in their group as only mild, homogeneity of their problem perception did not matter for successfully overcoming the problem and satisfaction (see Figure 3).

### 5.4 Qualitative analyses

Regarding the qualitative analysis, Group 55 (see Table 4) indicated a very homogeneous problem perception. All group members referred to a similar problem as the biggest problem, which they regarded as relatively severe. Consequently, they reported matching regulation strategies at different social levels. In the end, they assessed their biggest problem as solved and considered their collaboration as satisfactory.

**Table 4:** Case example Group 55.

Person (fictitious name)	Homogeneity	Biggest problem	Problem intensity	Biggest problem description	Self-regulation	Co-regulation (receiving support)	Co-regulation (giving support)	Socially shared regulation	Successful problem regulation	Satisfaction with the collaboration
Lisa	−0.23	Lack of technical functionality	4.00	Our group did not understand the software (Mind-Meister). The feature to create new bubbles and connect them with others did not work. We then switched to word, one edited the mind map and shared his screen. That worked faster and less complicated.	We noticed that we did not get along with the Mind-Meister program, so we agreed that one member of the group creates the mind map in a word document while sharing his screen. This was completely in line with my opinion.	Nothing, since we solved the problem together. We all had the same thoughts.	Since the problem with the software wasn’t due to the group members, we were all looking for a solution together. I did not need to think for others.	We noticed that we did not get along with the Mind-Meister program, so we agreed that one member of the group creates the mind map in a word document while sharing his screen.	5	5.0
Carina	−0.27	Lack of technical functionality	1.33	Designing the mind map together was complicated and complex.	NA	NA	NA	We created the mind map using word and only one person drew and wrote it.	5	3.4
Vanessa	−0.20	Lack of technical skills	2.00	As a group we had the difficulty of not being able to use the Mind-Meister website because the website did not offer any operating assistance.	I need to talk to the others about the problem and look for an alternative together.	One group member took over the drawing of the mind maps, so that it was easier for us as a group to continue working productively and leave the technical problems behind us.	I talked to the others about the technical difficulties and together we found an alternative.	One group member created the mind map on Word and shared her screen with us so that we could follow and discuss the creation of the mind map via Skype.	5	4.6

In contrast, group members from Group 74 (see Table 5) perceived their problems more differently. Furthermore, each member selected a different problem as the biggest problem. The first two group members assessed it as relatively weak. Their regulation seemed to be sufficient to overcome the problems, though the group as a whole was not engaged in regulating each problem. In comparison, the third group member reported an intense problem. As the group members’ statements depicted in Table 5 show, this problem was also not regulated by other group members or the group as a whole, and it was not regulated successfully. We will interpret these observations in the next section.

**Table 5:** Case example Group 74.

Person (fictitious name)	Homogeneity	Biggest problem	Problem intensity	Biggest problem description	Self-regulation	Co-regulation (receiving support)	Co-regulation (giving support)	Socially shared regulation	Successful problem regulation	Satisfaction with the collaboration
Sabrina	−0.57	Lack of technical functionality	1.67	The Mind-Meister program did not work as we had imagined.	We wrote down the mind map differently than we wanted to.	The others also tried to understand the program.	I tried to understand the program.	We tried to understand the program.	5	3.2
Nicolas	−0.85	Unclear task definition	1.00	At the beginning, the task was not entirely clear to me. However, this became clear while working on it. I only realized what should be on the map and why when I reread the task.	I read through the task step by step (before the group work) and formulated it with my own words. The previous emails with the instructions for the task were not clear to me at first, but the problem was then clarified without disturbing the group dynamic.	There was no assistance by my group members for better understanding. Didn’t explicitly ask for it either. Because the members understood the task well, I took even more time to understand it.	Not necessary. The problem was resolved before the start of the group phase.	Not necessary.	4	3.2
Alina	−1.13	Differing goals	3.00	Some group members were only aiming a quick completion of the work assignment, which also had a negative impact on the learning success of others.	I thought that I would take a closer look at the learning content for myself after the video conference so that I would at least remember some of the content. I would have wished for the content to be talked about and discussed more intensively, but I also did not want to hold the group back, since they—as I assume—wanted to be finished as quickly as possible, even though it then was done sloppily.	I went through parts of the presentation aloud with them, gave own examples, asked questions.	There’s no point in just making everything quick and wishy-washy.	It does not have to be perfect, unnecessary effort can be spared.	2	2.8

## 6 Discussion

This study investigated which problems occurred during a session of (relatively) self-organized online collaborative learning and how groups regulated these problems. Descriptive analyses of problem ratings and means of regulation success variables draw a picture of a rather successful learning experience: The problem each participant selected as their biggest problem had medium intensity only, and, at the same time, subjective measures of regulation success indicated successful regulation of these problems, high satisfaction and solid subjective knowledge gain. Overall, students seem to be prepared to successfully collaborate in this realm. At first glance, this finding contrasts with the results of Capdeferro and Romero (2012) who found students to report frustrations about online collaborative learning more frequently. A closer look at the concrete problems students reported to be the most intense reveals that technical issues were by far most often considered to be the biggest obstacles to collaborative learning. This mirrors findings from the literature which report the very same obstacles for whole courses or studying online during a whole semester (Belli, 2018; McCollum et al., 2019; Nungu et al., 2023; Rizvi and Nabi, 2021). Notions of Zoom fatigue (Fauville et al., 2023) in the literature might be represented in our data in the problem low value of the learning method which at least some of the students perceived. In contrast, problems regarding the communication, for example due to reduced visibility of non-verbal cues (Jeitziner et al., 2024) or social presence and trust (Bohannon et al., 2013; Qureshi et al., 2021; Shi et al., 2024), were not evident in our study. Maybe, the use of a digital visualization tool mitigated these potential pitfalls as Park et al. (2023) reported. Though lacking functionality or difficulties with using the mind-mapping software were the most reported problems, students seem to have regulated them successfully in most cases, for example, through switching to another tool. In general, the prevalence of problems specific to digital collaboration via videoconferencing indicates that students might indeed be less effective when they collaborate using a videoconferencing and a mind-mapping tool.

The main question of this study was how homogeneity of problem perceptions within study groups and immediacy and intensity of regulation strategy use would be associated with different measures of regulation success. In sum, homogeneity of problem perception was the only significant predictor of successful application of regulation strategies and satisfaction with the collaborative learning when moderated by the intensity of the biggest problem. If the problem was big, then a homogeneous perspective was associated with successful application of regulation strategies and satisfaction with the collaboration. If the problem was small, it did not matter how homogeneously the problems were perceived. This might mean that groups who have a commonly shared perspective on what their problems are are more successful in regulating their problems as soon as these problems become more severe. This finding is similar to the finding of Melzner et al. (2020).

The qualitative case examples illustrate this interaction effect and the potential mechanism behind it. In Group 55, an intense problem was seen homogeneously, regulated as a shared effort, and, therefore, overcome successfully. In Group 74, perspectives on what the main problem was differed more strongly. The group overcame the problems with low intensity nonetheless, but the problem with high intensity remained unresolved.

The comparison between these groups illustrates the interaction of homogeneity and problem intensity we found in the quantitative analyses. Perceiving the problems within a group homogeneously seems to be necessary to solve severe problems, but groups were able to solve mild problems without relying on a shared problem view. The case examples also shed light on the assumed mechanism driving this association between homogeneous problem perception and regulation success. We assumed that a homogeneous problem perception facilitates selecting regulation strategies (no matter if immediate or not) and executing them because it provides a common ground for all group members and a shared goal for regulation. This facilitation of regulation should matter especially when a problem is severe, that is, it challenges students’ resources to regulate it. Indeed, in the homogeneous Group 55, students regulated at different social levels all directed toward the same goal. In contrast, in the heterogeneous Group 74, students reported that they did not help each other to overcome the biggest problems they mutually perceived. There are two possible mechanisms explaining this lack of mutual help. It might be that the problem view was not shared; thus, no shared regulation regarding these problems developed because learners did not know about the problems other group members experienced. The low homogeneity values for this group and the statement of Alina that she is only assuming the other group members’ goals support this explanation. However, it might also be that the learners actually did know about other members’ problems but did not care enough to engage in respective co-regulation or socially shared regulation. Nonetheless, whatever mechanism was in place here, it resulted in that the severe problem had not been overcome.

Despite these significant effects regarding successful regulation of the biggest problem and satisfaction with the collaboration, subjective knowledge gain and objective knowledge were not associated with homogeneity of problem perceptions. The reason might be that students’ task to learn the lecture content was not a “real” group task including positive interdependence (Johnson and Johnson, 2009): An individual student could excel in this task even if the group fails to collaborate. Consequently, homogeneity might be associated only with variables concerning the collaboration directly, not the, in this case, very distal measures regarding knowledge gain.

Homogeneity regarding the social level at which the biggest problem was located was not significantly associated with any outcome variable. Seemingly, it did not matter for collaborative learning whether groups agreed on who was affected by the biggest problem. This might be explained as follows: A homogeneous perspective on the social localization of a problem means that students are sure who has a problem and who does not. However, to achieve this clarity, an explicit conversation about who is affected by a problem and to which extent might be necessary (Borge et al., 2018; Hadwin et al., 2018). Yet, to solve this problem, students need to focus on the content of the problem and provide solutions for it. Therefore, a conversation about who is affected exactly might—at least in this case—be a waste of time. In the end, groups might be better off if they focus on the content of the problem and thereby ignore the social localization of it. By doing so, homogeneous and heterogeneous groups (regarding problem localization) might equally effectively regulate a problem.

Contrary to Melzner et al. (2020), we did not find immediacy and intensity of strategy use to be associated with regulation success. This also contrasts with Engelschalk et al. (2016), who found strategies to be selectively used for different kinds of problems, but it is in line with Schoor and Bannert (2012), who also did not find an effect of intensity of regulation strategy use on regulation success. To better interpret this finding, it is informative to take the difference between this study and the study by Melzner et al. (2020) into account: Melzner et al. (2020) investigated completely self-organized groups preparing for important exams for an extended period of time, while the present study explored a single session of collaborative learning during a regular lecture. Thus, we compare an extensive, high stakes setting to a less extensive, lower stakes setting. In addition, the level of autonomy and instructional support differed: In Melzner et al. (2020), the learning content, materials, and method were completely self-selected, while in the present study, learning content, respective resources and materials, and some aspects of the learning method were fixed. In other words, in the present study, the instructional context might have helped to pave the way for collaborative learning enough, so that the specific strategy choice and intensity of its application did not matter for regulation success as much, because just any regulation strategy (applied with random intensity) might have been good enough to overcome a (rather) insignificant problem. In addition, students might have had more than usual practice with acquiring knowledge through studying digital learning material and videoconferencing in the first semester of online learning in the COVID-19 pandemic.

The fact that the instructional support in the present study seemed to be sufficient is slightly surprising though: When taking recommendations for instructional design of instances of collaborative learning (Strauß and Rummel, 2020) into account, only few principles were realized here. Strauß and Rummel derived these empirically supported principles from the literature to guide instructors to organize effective collaborative learning. “They greatly increase the probability that students will engage in beneficial interaction (Strauß and Rummel, 2020, p. 256).” Our realization of collaborative learning did not contain much learner support that makes a successful collaboration particularly likely. This was, of course, on purpose because we wanted to mimic the conditions of self-organized study groups. For example, we did not support students’ monitoring or script their interaction. Also, we did not design the task to create positive interdependence and did not adapt the level of complexity (all recommendations for effective instructional design from Strauß and Rummel, 2020). Instead, the task was designed to mirror the goal of self-organized study groups who usually have the goal to understand and memorize a given subject matter for an exam. For these reasons, we had to expect problems to occur similarly to self-organized collaborative studying and for learning to be less effective than with optimal instructional support.

The same is true for the technical realization: Only three out of seven affordances for computer supported collaborative learning that were proposed by Jeong and Hmelo-Silver (2016) were used here (video chat as communication means, concept map as representational tool, and facilitation of group formation). This constitutes only a basis for an interaction to happen, but it does not provide technological support for high-quality interaction. For example, sharing of not commonly shared knowledge was not encouraged, structuring the interaction was not enhanced, and monitoring and regulation was not supported by technology (Jeong and Hmelo-Silver, 2016). Therefore, it was likely that common problems of ineffective collaboration such as free riding, a lack of transactive dialogue (Vogel et al., 2016), and, consequently, an only superficial processing of the subject matter may have occurred.

These concerns are corroborated by considering what we know for sure regarding which concrete actions students performed themselves. As the instructional and technological design did not particularly encourage students to engage in high-quality interaction, it remains unclear if students applied more than two strategies out of 10 (MacMahon et al., 2020), namely scheduling uninterrupted work and creating a shared concept map, which the authors recommended to students for effective learning.

In summary, we provided only very simple instructional and technical support (basically only initiating the collaboration and demanding a visualization) that distinguished the current setting from truely self-organized studying with zero instructional guidance. Nonetheless and surprisingly, our findings indicated that students did not need to employ the theoretically most beneficial regulation (i.e., immediate) strategies and to use strategies with sufficient intensity to succeed in this collaborative learning. This may mean that a low-level instructional support already makes a big difference and helps to simplify the dynamics of self-organized collaborative learning in a way that students cope successfully with upcoming problems.

We conclude that the full model of problem regulation shown in Figure 1 might only apply to truly self-organized learning contexts with sufficient prevalence of problems, while problem regulation might follow a simpler process that involves only relying on a shared problem perception when problems are low due to effective instructional support. Yet, further research is needed to test this interpretation.

## 7 Limitations

When interpreting the results, we have to take the following limitations into account. First, neither the predictor variables nor the subjective measures of regulation success were associated with the results of the objective knowledge test. In addition to the explanation regarding the nature of the task discussed above, there are several further possible explanations for this: It might be that the actual knowledge is influenced by many other variables not in the scope of this study which might increase unsystematic error variance, making it difficult to find small effects. Alternatively, the lack of a significant association might be due to the low prevalence of problems which might have created a ceiling effect, therefore reducing variance and possible covariation. Previous research indicated that groups dedicate only a small amount of a session of collaborative learning to regulation of problems (Nguyen et al., 2023). The vast majority is available to focus on the subject matter. Therefore, it is unlikely that regulation has a large impact on learning gains as long as the problem intensity is generally low. Furthermore, a lack of validity of the test might also be responsible for the lack of associations with other variables in this study. We have no data on validity of the test beyond item analyses and our careful mapping of learning material content to test items to test this possibility.

Second, all measures (except the knowledge test) were based on self-report, though regulation strategies were measured by open-ended questions at least in order to reduce social desirability bias. True associations might be different.

Third, localizing the biggest problem at different social levels might be difficult for students. Especially, it might be hard to guess how much others were affected by a problem. Therefore, our measurement might include a lot of random variation which obscures any potential effect.

Fourth, we operationalized regulation intensity using the number of strategies which students mentioned. However, this represents the construct of intensity only partly. In principle, learners could have exhibited a single strategy very intensely, too. Prior research mostly used only frequency of regulation strategies as indicator of regulation intensity (Cumming, 2010; Schwinger et al., 2009; Su et al., 2018). Though, in principle, intensity might be measured by how often and how long students tried a certain strategy, and how much effort they invested to try to make that strategy work. Therefore, a more comprehensive measurement of intensity should include both aspects, number of strategies and implementation frequency, duration, and effort for each strategy. Such a measurement of intensity might yield stronger effects on regulation or learning success.

Fifth, the study took place in the first semester following the onset of the COVID-19 pandemic. At that time, students might not have been as familiar with videoconferencing as they are now, although the study was more at the end of the semester. This might explain some of the technical difficulties students had, qualifying this part of the descriptive findings presented in Figure 2.

Sixth, descriptive results of problem intensity might be biased. In principle, it is possible that groups who encountered severe problems broke off from the collaboration and did not answer the questionnaire afterwards. Therefore, especially the most severe problems might not be represented in the data. Unfortunately, we have no data to check whether this was the case. We can only say that the available rating scale was used to full extent (see Figure 2).

Seventh, we do not know how well group members knew each other or whether they already worked together in other courses. In other studies, subjective outcomes are fostered by group members familiarity with each other (Crompton et al., 2022; Janssen et al., 2009; Zhang et al., 2023a; Zhang et al., 2023b), whereas findings regarding objective performance are less conclusive and often find no effect (Crompton et al., 2022; Janssen et al., 2009). As the two lectures were large and students came from many different subjects, we assume that a considerable proportion of group members might have been unfamiliar with each other. However, with our data, we cannot determine to which extent familiarity with each other moderates our findings.

Eighth, our findings should not be generalized to other group sizes. In our study, group sizes ranged from two to three. These group sizes should reflect typical sizes of self-organized study groups. Larger groups might suffer from more coordination issues and a higher likelihood of free-riding and other phenomena indicating a reduced individual engagement. However, research draws a differentiated picture of an optimal group size (Wang et al., 2023), favoring either two, three, or four people per group. Therefore, groups with four or more students might function differently.

## 8 Implications

The interpretation of the differences between the findings in the previous study by Melzner et al. (2020) and the results in the present study has important implications for theory building: A new theoretical model of problem regulation during collaborative learning needs to be developed that (a) includes problem intensity as a moderator of the relations between problems, their regulation, and learning outcome, and (b) takes the context regarding its incentive structure (i.e., low vs. high stakes) into account. For teaching practice, the study might imply that recommendations of good instructional design for collaborative learning (see above) also apply to relatively self-organized online collaborative learning and that simple and few scaffolding aids might already help to reach satisfying collaboration success.

## Data Availability

The dataset presented in this study can be found in the online repository Open Science Framework at: https://osf.io/68by5/.

## References

[ref1] Al-Samarraie H. Saeed N. (2018). A systematic review of cloud computing tools for collaborative learning: opportunities and challenges to the blended-learning environment. Comput. Educ. 124, 77–91. doi: 10.1016/j.compedu.2018.05.016

[ref2] Artelt C. Beinicke A. Schlagmüller M. Schneider W. (2009). Diagnose von Strategiewissen beim Textverstehen [Assessing knowledge about reading strategies]. Zeitschrift für Entwicklungspsychologie und Pädagogische Psychologie 41, 96–103. doi: 10.1026/0049-8637.41.2.96

[ref3] Atkinson R. C. Shiffrin R. M. (1968). “Human memory: a proposed system and its control processes” in The psychology of learning and motivation: advances in research and theory. eds. Spence K. W. Spence J. T., vol. 2 (New York: Academic Press), 89–197.

[ref4] Bates D. Mächler M. Bolker B. Walker S. (2015). Fitting linear mixed-effects models using lme4. J. Stat. Softw. 67, 1–48. doi: 10.18637/jss.v067.i01

[ref5] Bäulke L. Eckerlein N. Dresel M. (2018). Interrelations between motivational regulation, procrastination and college dropout intentions. Unterrichtswissenschaft 46, 461–479. doi: 10.1007/s42010-018-0029-5

[ref6] Bellhäuser H. Müller A. Konert J. Röpke R. (2019). “Birds of a feather learn well together? An experimental study on the effect of homogeneous and heterogeneous learning group composition on satisfaction and performance” in A wide lens: combining embodied, enactive, extended, and embedded learning in collaborative settings, 13th international conference on computer supported collaborative learning (CSCL). eds. Lund K. Niccolai G. Lavoué E. Hmelo-Silver C. Gweon G. Baker M., vol. 2 (Lyon: International Society of the Learning Sciences), 721–722. Available at: https://repository.isls.org//handle/1/4496

[ref7] Belli S. (2018). Managing negative emotions in online collaborative learning: a multimodal approach to solving technical difficulties. Digithum 22, 35–46. doi: 10.7238/d.v0i22.3140

[ref8] Bohannon L. S. Herbert A. M. Pelz J. B. Rantanen E. M. (2013). Eye contact and video-mediated communication: a review. Displays 34, 177–185. doi: 10.1016/j.displa.2012.10.009

[ref9] Borge M. Ong Y. S. Rosé C. P. (2018). Learning to monitor and regulate collective thinking processes. Int. J. Comput.-Support. Collab. Learn. 13, 61–92. doi: 10.1007/s11412-018-9270-5

[ref10] Camilleri M. A. Camilleri A. C. (2022). Remote learning via video conferencing technologies: implications for research and practice. Technol. Soc. 68:101881. doi: 10.1016/j.techsoc.2022.101881, PMID: 35034998 PMC8743284

[ref11] Capdeferro N. Romero M. (2012). Are online learners frustrated with collaborative learning experiences? Int. Rev. Res. Open Distrib. Learn. 13, 26–44. doi: 10.19173/irrodl.v13i2.1127

[ref12] Chen J. Wang M. Kirschner P. A. Tsai C.-C. (2018). The role of collaboration, computer use, learning environments, and supporting strategies in CSCL: a meta-analysis. Rev. Educ. Res. 88, 799–843. doi: 10.3102/0034654318791584

[ref13] Chi M. T. H. Wylie R. (2014). The ICAP framework: linking cognitive engagement to active learning outcomes. Educ. Psychol. 49, 219–243. doi: 10.1080/00461520.2014.965823

[ref14] Crompton C. J. Wolters M. K. MacPherson S. E. (2022). Learning with friends and strangers: partner familiarity does not improve collaborative learning performance in younger and older adults. Memory 30, 636–649. doi: 10.1080/09658211.2022.2041038 35193481

[ref15] Cumming J. (2010). Student-initiated group management strategies for more effective and enjoyable group work experiences. J. Hosp. Leisure Sport Tour. 9, 31–45. doi: 10.3794/johlste.92.284

[ref16] Eckerlein N. Roth A. Engelschalk T. Steuer G. Schmitz B. Dresel M. (2019). The role of motivational regulation in exam preparation: results from a standardized diary study. Front. Psychol. 10:81. doi: 10.3389/fpsyg.2019.00081, PMID: 30804828 PMC6370677

[ref17] Engelschalk T. Steuer G. Dresel M. (2016). Effectiveness of motivational regulation: dependence on specific motivational problems. Learn. Individ. Differ. 52, 72–78. doi: 10.1016/j.lindif.2016.10.011

[ref18] Fauville G. Luo M. Queiroz A. C. M. Lee A. Bailenson J. N. Hancock J. (2023). Video-conferencing usage dynamics and nonverbal mechanisms exacerbate zoom fatigue, particularly for women. Comput. Hum. Behav. Rep. 10:100271. doi: 10.1016/j.chbr.2023.100271

[ref19] Fett K. Greisel M. Dresel M. Kollar I. (2021). Do all roads lead to Rome? An expert study to assess the immediacy of strategies to regulate collaborative learning. In Hmelo-Silver C. E. Wever B. De Oshima J. (Eds.), Proceedings of the 14th international conference on computer-supported collaborative learning—CSCL 2021 (pp. 201–204). International Society of the Learning Sciences.

[ref20] Glaesmer H. Grande G. Braehler E. Roth M. (2011). The German version of the satisfaction with life scale (SWLS). Psychometric properties, validity, and population-based norms. Eur. J. Psychol. Assess. 27, 127–132. doi: 10.1027/1015-5759/a000058

[ref9001] Greisel M. Melzner N. Kollar I. Dresel M. (2023). How are achievement goals associated with self-, co-, and socially shared regulation in collaborative learning? Educ. Psychol. 43, 384–402. doi: 10.1080/01443410.2023.2211751

[ref21] Hadwin A. F. Järvelä S. Miller M. (2018). “Self-regulation, co-regulation, and shared regulation in collaborative learning environments” in Handbook of self-regulation of learning and performance. eds. Schunk D. H. Zimmerman B.. 2nd ed (New York: Routledge/Taylor & Francis Group), 83–106.

[ref22] Janssen J. Erkens G. Kirschner P. A. Kanselaar G. (2009). Influence of group member familiarity on online collaborative learning. Comput. Hum. Behav. 25, 161–170. doi: 10.1016/j.chb.2008.08.010

[ref23] Järvelä S. Hadwin A. F. (2013). New frontiers: regulating learning in CSCL. Educ. Psychol. 48, 25–39. doi: 10.1080/00461520.2012.748006

[ref24] Järvenoja H. Näykki P. Törmänen T. (2019). Emotional regulation in collaborative learning: when do higher education students activate group level regulation in the face of challenges? Stud. High. Educ. 44, 1747–1757. doi: 10.1080/03075079.2019.1665318

[ref25] Järvenoja H. Volet S. Järvelä S. (2013). Regulation of emotions in socially challenging learning situations: an instrument to measure the adaptive and social nature of the regulation process. Educ. Psychol. 33, 31–58. doi: 10.1080/01443410.2012.742334

[ref26] Jeitziner L. T. Paneth L. Rack O. Zahn C. (2024). Beyond words: investigating nonverbal indicators of collaborative engagement in a virtual synchronous CSCL environment. Front. Psychol. 15:1347073. doi: 10.3389/fpsyg.2024.1347073 39205972 PMC11349685

[ref27] Jeong H. Hmelo-Silver C. E. (2016). Seven affordances of computer-supported collaborative learning: how to support collaborative learning? How can technologies help? Educ. Psychol. 51, 247–265. doi: 10.1080/00461520.2016.1158654

[ref28] Jeong H. Hmelo-Silver C. E. Jo K. (2019). Ten years of computer-supported collaborative learning: a meta-analysis of CSCL in STEM education during 2005–2014. Educ. Res. Rev. 28:100284. doi: 10.1016/j.edurev.2019.100284

[ref29] Johnson D. W. Johnson R. T. (2009). An educational psychology success story: social interdependence theory and cooperative learning. Educ. Res. 38, 365–379. doi: 10.3102/0013189X09339057

[ref30] Kirschner F. Paas F. Kirschner P. A. (2009). A cognitive load approach to collaborative learning: united brains for complex tasks. Educ. Psychol. Rev. 21, 31–42. doi: 10.1007/s10648-008-9095-2

[ref31] Kirschner F. Paas F. Kirschner P. A. Janssen J. (2011). Differential effects of problem-solving demands on individual and collaborative learning outcomes. Learn. Instr. 21, 587–599. doi: 10.1016/j.learninstruc.2011.01.001

[ref32] Koivuniemi M. Panadero E. Malmberg J. Järvelä S. (2017). Higher education students’ learning challenges and regulatory skills in different learning situations. J. Study Educ. Dev. 40, 19–55. doi: 10.1080/02103702.2016.1272874

[ref33] Kuznetsova A. Brockhoff P. B. Christensen R. H. B. (2017). lmerTest package: tests in linear mixed effects models. J. Stat. Softw. 82, 1–26. doi: 10.18637/jss.v082.i13

[ref34] Kyndt E. Raes E. Lismont B. Timmers F. Cascallar E. Dochy F. (2013). A meta-analysis of the effects of face-to-face cooperative learning. Do recent studies falsify or verify earlier findings? Educ. Res. Rev. 10, 133–149. doi: 10.1016/j.edurev.2013.02.002

[ref35] MacMahon S. Leggett J. Carroll A. (2020). Promoting individual and group regulation through social connection: strategies for remote learning. Inf. Learn. Sci. 121, 353–363. doi: 10.1108/ILS-04-2020-0101

[ref36] Malmberg J. Järvelä S. Järvenoja H. Panadero E. (2015). Promoting socially shared regulation of learning in CSCL: progress of socially shared regulation among high- and low-performing groups. Comput. Hum. Behav. 52, 562–572. doi: 10.1016/j.chb.2015.03.082

[ref9002] McCollum B. Morsch L. Shokoples B. Skagen D. (2019). Overcoming barriers for implementing international online collaborative assignments in chemistry. Can. J. Scholarsh. Tea. 10:6. doi: 10.5206/cjsotl-rcacea.2019.1.8004

[ref37] Melzner N. Greisel M. Dresel M. Kollar I. (2020). Regulating self-organized collaborative learning: the importance of homogeneous problem perception, immediacy and intensity of strategy use. Int. J. Comput.-Support. Collab. Learn. 15, 149–177. doi: 10.1007/s11412-020-09323-5, PMID: 32837406 PMC7328647

[ref38] Nguyen A. Järvelä S. Rosé C. Järvenoja H. Malmberg J. (2023). Examining socially shared regulation and shared physiological arousal events with multimodal learning analytics. Br. J. Educ. Technol. 54, 293–312. doi: 10.1111/bjet.13280

[ref39] Nokes-Malach T. J. Richey J. E. Gadgil S. (2015). When is it better to learn together? Insights from research on collaborative learning. Educ. Psychol. Rev. 27, 645–656. doi: 10.1007/s10648-015-9312-8

[ref40] Noroozi O. Bayat A. Hatami J. (2019). “Effects of a digital guided peer feedback system on student learning and satisfaction” in A wide lens: combining embodied, enactive, extended, and embedded learning in collaborative settings, 13th international conference on computer supported collaborative learning (CSCL). eds. Lund K. Niccolai G. Lavoué E. Hmelo-Silver C. Gweon G. Baker M., *Vol*. 2 (Lyon: International Society of the Learning Sciences), 809–810. Available at: https://repository.isls.org//handle/1/4508

[ref41] Nungu L. Mukama E. Nsabayezu E. (2023). Online collaborative learning and cognitive presence in mathematics and science education. Case study of University of Rwanda, College of Education. Educ. Inf. Technol. 28, 10865–10884. doi: 10.1007/s10639-023-11607-w, PMID: 36779197 PMC9905761

[ref42] Park K. Farb A. George B. (2023). Effectiveness of visual communication and collaboration tools for online GIS teaching: using Padlet and Conceptboard. J. Geogr. High. Educ. 47, 399–410. doi: 10.1080/03098265.2022.2065669

[ref43] Qureshi M. A. Khaskheli A. Qureshi J. A. Raza S. A. Yousufi S. Q. (2021). Factors affecting students’ learning performance through collaborative learning and engagement. Interact. Learn. Environ. 31, 2371–2391. doi: 10.1080/10494820.2021.1884886

[ref44] R Core Team (2022). R: a language and environment for statistical computing. Vienna, Austria: R Foundation for Statistical Computing. Available at: https://www.R-project.org/

[ref45] Ritzmann S. Hagemann V. Kluge A. (2014). The training evaluation inventory (TEI)—evaluation of training design and measurement of training outcomes for predicting training success. Vocat. Learn. 7, 41–73. doi: 10.1007/s12186-013-9106-4

[ref46] Rizvi Y. S. Nabi A. (2021). Transformation of learning from real to virtual: an exploratory-descriptive analysis of issues and challenges. J. Res. Innov. Teach. Learn. 14, 5–17. doi: 10.1108/JRIT-10-2020-0052

[ref47] Schoor C. Bannert M. (2012). Exploring regulatory processes during a computer-supported collaborative learning task using process mining. Comput. Hum. Behav. 28, 1321–1331. doi: 10.1016/j.chb.2012.02.016

[ref48] Schwinger M. Steinmayr R. Spinath B. (2009). How do motivational regulation strategies affect achievement: mediated by effort management and moderated by intelligence. Learn. Individ. Differ. 19, 621–627. doi: 10.1016/j.lindif.2009.08.006

[ref49] Shaw J. D. Duffy M. K. Stark E. M. (2000). Interdependence and preference for group work: main and congruence effects on the satisfaction and performance of group members. J. Manag. 26, 259–279. doi: 10.1177/014920630002600205

[ref50] Shi Y. Cheng Q. Wei Y. Tong M. Yao H. (2024). Understanding the effect of video conferencing learning environments on students’ engagement: the role of basic psychological needs. J. Comput. Assist. Learn. 40, 288–305. doi: 10.1111/jcal.12880

[ref51] Splichal J. M. Oshima J. Oshima R. (2018). Regulation of collaboration in project-based learning mediated by CSCL scripting reflection. Comput. Educ. 125, 132–145. doi: 10.1016/j.compedu.2018.06.003

[ref52] Springer L. Stanne M. E. Donovan S. S. (1999). Effects of small-group learning on undergraduates in science, mathematics, engineering, and technology: a meta-analysis. Rev. Educ. Res. 69, 21–51. doi: 10.3102/00346543069001021

[ref53] Steuer G. Engelschalk T. Eckerlein N. Dresel M. (2019). Assessment and relationships of conditional motivational regulation strategy knowledge as an aspect of undergraduates’ self-regulated learning competencies. Zeitschrift Für Pädagogische Psychologie 33, 95–104. doi: 10.1024/1010-0652/a000237

[ref54] Strauß S. Rummel N. (2020). Promoting interaction in online distance education: designing, implementing and supporting collaborative learning. Inf. Learn. Sci. 121, 251–260. doi: 10.1108/ILS-04-2020-0090

[ref55] Su Y. Li Y. Hu H. Rosé C. P. (2018). Exploring college English language learners’ self and social regulation of learning during wiki-supported collaborative reading activities. Int. J. Comput. Support. Collab. Learn. 13, 35–60. doi: 10.1007/s11412-018-9269-y

[ref56] Tan C. Casanova D. Huet I. Alhammad M. (2022). Online collaborative learning using Microsoft teams in higher education amid COVID-19. Int. J. Mobile Blended Learn. 14, 1–18. doi: 10.4018/IJMBL.297976

[ref57] Vogel F. Kollar I. Ufer S. Reichersdorfer E. Reiss K. Fischer F. (2016). Developing argumentation skills in mathematics through computer-supported collaborative learning: the role of transactivity. Instr. Sci. 44, 477–500. doi: 10.1007/s11251-016-9380-2

[ref58] Waldeyer J. Fleischer J. Wirth J. Leutner D. (2019). Entwicklung und erste Validierung eines situational-judgement-instruments zur Erfassung von Kompetenzen im Bereich des Ressourcenmanagements (ReMI) [Development and validation of a new instrument for students’ resource management assessment]. Diagnostica 65, 108–118. doi: 10.1026/0012-1924/a000217

[ref9003] Wang M. Jiang L. Luo H. (2023). Dyads or quads? Impact of group size and learning context on collaborative learning. Front. psychol. 14:1168208. doi: 10.3389/fpsyg.2023.1168208 37213364 PMC10196372

[ref59] Wecker C. Fischer F. (2014). “Lernen in Gruppen [Learning in groups]” in Pädagogische Psychologie. eds. Seidel T. Krapp A.. 6th ed (Weinheim: Beltz), 277–296.

[ref60] Weinberger A. Stegmann K. Fischer F. (2012). Learning to argue online: scripted groups surpass individuals (unscripted groups do not). Comput. Hum. Behav. 26, 506–515. doi: 10.1016/j.chb.2009.08.007

[ref61] Winne P. H. Hadwin A. F. (1998). “Studying as self-regulated engagement in learning” in Metacognition in educational theory and practice. eds. Hacker D. Dunlosky J. Graesser A. (Hillsdale, NJ: Erlbaum), 277–304.

[ref62] Zhang S. Che S. Nan D. Li Y. Kim J. H. (2023a). I know my teammates: the role of group member familiarity in computer-supported and face-to-face collaborative learning. Educ. Inf. Technol. 28, 12615–12631. doi: 10.1007/s10639-023-11704-w, PMID: 37361813 PMC10009824

[ref63] Zhang S. Nan D. Sun S. Che S. Kim J. H. (2023b). Teammate familiarity in distributed computer-supported collaborative learning: the mediating role of social presence. Int. Rev. Res. Open Distrib. Learn. 24, 233–251. doi: 10.19173/irrodl.v24i4.7332

[ref64] Zimmermann B. J. Moylan A. R. (2009). “Self-regulation: where metacognition and motivation intersect” in Handbook of metacognition in education. eds. Hacker D. J. Dunlosky J. Graesser A. C. (New York: Routledge), 299–315. Available at: 10.4324/9780203876428

